# ISOLATION AND TRANSCRIPTOME ANALYSIS OF ADULT ZEBRAFISH CELLS ENRICHED FOR SKELETAL MUSCLE PROGENITORS

**DOI:** 10.1002/mus.21972

**Published:** 2011-02-17

**Authors:** Matthew S Alexander, Genri Kawahara, Alvin T Kho, Melanie H Howell, Timothy J Pusack, Jennifer A Myers, Federica Montanaro, Leonard I Zon, Jeffrey R Guyon, Louis M Kunkel

**Affiliations:** 1 Program in Genomics and Howard Hughes Medical Institute, Center for Life Science, Room CLS15027.1, Children's Hospital Boston3 Blackfan Circle, Boston, Massachusetts 02115, USA; 2 Stem Cell Program and Hematology/Oncology and Howard Hughes Medical Institute, Children's Hospital BostonBoston, Massachusetts, USA; 3 Auke Bay Laboratories, Alaska Fisheries Science Center, NOAA FisheriesJuneau, Alaska, USA; 4 Research Institute at Nationwide Children's Hospital, Department of Pediatrics, Center in Gene Therapy, Ohio State University College of MedicineColumbus, Ohio, USA; 5 Manton Center for Orphan Disease Research, Children's Hospital BostonBoston, Massachusetts, USA; 6 Harvard Stem Cell InstituteCambridge, Massachusetts, USA

**Keywords:** differentiation, muscle mutants, muscle stem cells, myogenic progenitors, myogenesis, transcriptome, zebrafish

## Abstract

Introduction: Over the past 10 years, the use of zebrafish for scientific research in the area of muscle development has increased dramatically. Although several protocols exist for the isolation of adult myoblast progenitors from larger fish, no standardized protocol exists for the isolation of myogenic progenitors from adult zebrafish muscle. Methods: Using a variant of a mammalian myoblast isolation protocol, zebrafish muscle progenitors have been isolated from the total dorsal myotome. These zebrafish myoblast progenitors can be cultured for several passages and then differentiated into multinucleated, mature myotubes. Results: Transcriptome analysis of these cells during myogenic differentiation revealed a strong downregulation of pluripotency genes, while, conversely, showing an upregulation of myogenic signaling and structural genes. Conclusions: Together these studies provide a simple, yet detailed method for the isolation and culture of myogenic progenitors from adult zebrafish, while further promoting their therapeutic potential for the study of muscle disease and drug screening. *Muscle Nerve* **43**: 741–750, 2011

The use of zebrafish (*Brachydanio rerio*) as an animal model for scientific research has dramatically increased in recent years as more researchers recognize its value in elucidating molecular pathways in normal and diseased states.^1^ The ability to knock down gene expression in embryos using morpholinos has allowed researchers to perform reverse genetics to identify genes essential for vertebrate development in a high-throughput fashion. In addition, the zebrafish is an excellent model to study early myogenesis in an ex utero setting, which allows for the molecular determination of the timing events in somatogenesis that occur from the initial somites to mature myofibers.^2^, ^3^ Over the past few years, several zebrafish skeletal muscle mutants with links to human myopathies and dystrophies have been identified, such as *sapje* and *sapje-like* (dystrophin), *runzel* (titin), *softy* (laminin β2), and *candyfloss* (laminin α2), and have provided valuable insight into the progression of muscle disease.^4^–^8^ The high degree of evolutionary conservation of myogenesis between mammals and zebrafish renders loss-of-function (morpholinos) or gain-of-function (transgenic fish) experimentation both economical and rapid.^9^ Consequently, the development of an efficient and simple method for the isolation and in vitro study of myogenic progenitors from adult zebrafish muscle mutants, combined with the amenability of zebrafish for high-throughput chemical screens, can significantly accelerate identification of compounds and optimization of parameters for new therapeutic approaches prior to further evaluation in mammalian disease models.

There are many approaches for treating muscular dystrophies and myopathies. Cell-based therapy is among the more promising options.^10^ For cell therapy, therapeutic cells are transferred to the host recipient to treat the cause or symptoms of the disease. Recent experiments in mouse transgenic models have focused on enriching for cells with myogenic potential in the hopes that these cells will be able to successfully engraft and correct the disease. The molecular pathways involved in early zebrafish myogenesis have been shown to share a large amount of evolutionary conservation with that of the more well-characterized mouse animal model.^11^ Recent advances in zebrafish myogenesis have demonstrated that blastomeres isolated from zebrafish embryos can be transduced into myogenic cell cultures with the addition of hedgehog.^12^ Further experiments in larger fish species, such as the Atlantic salmon (*Salmo salar*) and the rainbow trout (*Oncorhynchus mykiss*), have resulted in the successful isolation, differentiation, and molecular characterization of adult dorsal myotome myoblasts grown in cell culture.^13^, ^14^

Despite the increased interest in the use of zebrafish to study muscle development and disease, no current protocol exists for the successful isolation and characterization of myogenic progenitors from the adult zebrafish skeletal muscle.^15^ Using a variant method from mammalian myoblast progenitor cell isolation,^16^ we have successfully isolated zebrafish myogenic progenitor cells from adult zebrafish whole dorsal myotome muscle. Utilizing an α-actin–red flourescent protein (RFP) transgenic fish line that expresses RFP exclusively in skeletal muscle, these adult zebrafish myogenic progenitor cells have been expanded and differentiated into multinucleated myotubes. Microarray transcriptome data obtained from these cells taken at critical time-points during myogenic differentiation have revealed a clustering of downregulated myogenic pluripotency markers (pax7a, myf5, ncam1a, etc.), but strong upregulation of myogenic signaling and structural genes (desmin, caveolin-3, α-actin-1a, etc). Together, these experiments provide a simple, yet concise method for the isolation of adult zebrafish skeletal myoblast progenitors from whole dorsal myotome muscle, which greatly expands zebrafish utility for in vitro cell culture differentiation experiments, myoblast transplantation, and chemical screening for novel drug-based therapies in muscle mutants.

## METHODS

### Fish Lines

The α-actin–RFP transgenic fish line was a generous gift from H.J. Tsai (Taiwan National University) and has been described previously.^17^ Additional experiments were done utilizing the wild-type AB strain, which was obtained from the Children's Hospital aquatics program and maintained in their aquatics facility. All animal protocols were approved by the animal resources committee of Children's Hospital.

### Isolation of Zebrafish Myogenic Muscle Cells from Whole Dorsal Myotome

For each cell preparation, 15–20 adult zebrafish were euthanized in tricaine (Sigma-Aldrich) and the whole zebrafish was placed in 100% ethanol for 30 seconds as the first step for sterilization. The fish's head, tail, and fins were removed with a scalpel, and the skin and internal organs were removed with forceps. The fish's body was sterilized in 10% bleach for 30 seconds and then washed twice in sterile phosphate-buffered saline (PBS) for another 30 seconds. Fish dorsal muscle and bone were minced with a scalpel and then transferred to a pre-weighed culture plate. For every gram of fish tissue, 3.5 ml of collagenase IV (10 mg/ml stock solution) and 3.5 ml of dispase (2.4 units/ml stock solution; Worthington Chemicals) were added and mixed by pipetting (Worthington). The solution was incubated at room temperature for 45 minutes (mixed every 10 minutes by pipette) before 10 ml of growth medium (L15; Sigma-Aldrich), 3% fetal calf serum, 100 μg/ml penicillin/streptomycin, 2 mM glutamine, and 0.8 mM CaCl_2_ (all Sigma-Aldrich) were added to the cells to quench the activity of the collagenase and dispase proteases. Debris was removed by filtering the cells through a 70-μm filter and then through two 40-μm filters (BD Biosciences). On each occasion, the filters were washed with 5 ml of L15 medium.

The cells were isolated by centrifugation at 1000 × *g* for 10 minutes at 9°C, and the supernatant was aspirated. The cells were then resuspended in 3 ml of red blood cell lysis buffer (Qiagen) and incubated for 3 minutes at room temperature before neutralization with 22 ml of L15 growth medium. The cells were then pelleted at 1000 × *g* for 10 minutes at 9°C, the supernatant aspirated, and the cell pellet resuspended in 3 ml of cold 1× PBS and layered on top of 4 ml of Ficoll-Paque gradient (GE Healthcare) in a 15-ml tube. Samples were then centrifuged at 1400 × *g* for 40 minutes at 9°C. A mononuclear cell layer was then extracted by pipette and washed with 10 ml of ice-cold 1× PBS. Afterwards, the cells were resuspended in 10 ml of ice-cold L15 buffer. The cell density was determined using an automated hemocytometer (Countess; Invitrogen), and the cell suspension was diluted in L15 growth medium.

The cells were then pre-plated on uncoated plates for 1 hour in a 28°C tissue culture incubator at 5% CO_2_. After pre-plating, the cellular supernatant (non-adherent cells) was removed and placed on laminin-coated plates (BD Biocoat). Alternatively, 0.1% gelatin-coated (porcine) plates can be used. The medium was changed every 3 days. The zebrafish myogenic progenitor cells were able to be grown for up to seven doublings before evidence of cellular senescence, with an average of four and five doublings per myoblast isolation. On average, a yield of 5–10 million live (trypan blue–negative) cells were isolated from each preparation of between 15 and 20 adult zebrafish. Lower yields of 100,000–500,000 live cells were isolated when using 1–5 adult zebrafish.

An alternative to the L15 growth medium was later used in zebrafish myogenic progenitor cell cultures and achieved the same results. Human skeletal myoblast growth medium (Promocell) that contained 20% fetal bovine serum (Atlanta Biologicals), 1× antibiotic–antimycotic (Invitrogen), and 1× Glutamax (Invitrogen), and supplemented with 3 ng/ml recombinant human fibroblast-like growth factor (rhFGF; Promega), can be used in lieu of the L15 growth medium.

### Myogenic Differentiation of Adult Zebrafish Myogenic Progenitor Cells

Approximately 300,000 cells/well were plated into six-well 0.1% gelatin-coated plates in 2 ml of growth medium and grown to 95% confluence. The medium was then changed to differentiation medium consisting of: 2% horse serum (Gibco) in Dulbecco modified Eagle medium (DMEM; Mediatech, Inc.) supplemented with 1× antibiotic–antimycotic (Invitrogen) and 1× Glutamax (Invitrogen). The differentiation medium was changed every other day, and cells were monitored for myotube fusion by phase and fluorescent microscopy. Multinucleated myotubes were observed during days 4–7.

### Immunohistochemistry

The following primary antibodies were used for immunohistochemistry of zebrafish myogenic progenitor cells: Pax3 mouse monoclonal (1:25; Developmental Studies Hybridoma Bank); Pax7 mouse monoclonal (1:25; Developmental Studies Hybridoma Bank); anti-MyoD1 rabbit polyclonal (1:50; Santa Cruz Biotechnology); and anti-myogenin rabbit polyclonal (1:50; M-225; Santa Cruz Biotechnology). The myogenin antibody has been characterized previously in early zebrafish myogenic progenitor cells.^18^ The zebrafish myod1 epitope has been shown to be recognized by the myf5 antibody (Santa Cruz Biotechnology).^19^

Approximately, 100,000 cells were pre-plated on uncoated coverslides (Nunc, Lab-Tek) and, after a 1-hour pre-plating, the supernatant was plated onto 0.1% gelatin-collated coverslips. The following day, the zebrafish myogenic cells attached were fixed in 4% paraformaldehyde (Electron Microscopy Sciences) at 4°C for 10 minutes. To block nonspecific binding of the antibodies, slides were incubated for 30 minutes at room temperature in PBS + 10% goat serum. After blocking, the slides were incubated overnight at 4°C using the primary antibodies. Slides were washed three times in 1× PBS, and sections were incubated with Alexa 488 (anti-mouse IgG)- or 568 (anti-rabbit IgG)-conjugated goat secondary antibodies (Invitrogen) at a 1:500 dilution for 45 minutes at room temperature. The slides were then washed three times in 1× PBS before mounting in Vectashield with 4′,6-diamidino-2-phenylindole (DAPI) (Vector Laboratories). Slides were analyzed by microscope (E1000 Nikon Eclipse; Nikon) and OpenLab software.

### RNA Isolation and Microarray Analysis

RNA was extracted directly from zebrafish myogenic progenitor cells in culture at various stages of differentiation using Tripure (Roche Applied Science), following the manufacturer's protocol. Zebrafish cDNA was hybridized to the Affymetrix GeneChip Zebrafish Genome Array (GenBank Release 36.0, June 2003) and processed following the manufacturer's protocol at the Molecular Genetics Core Facility at Children's Hospital Boston. The resulting. CEL files, which contain probe signal intensities of the samples, were preprocessed and normalized together using robust multiarray averaging (RMA), which returns the expression level of each probe set or gene as a positive real number in logarithmic base 2 scale.^20^ The complete microarray data are available from the NCBI Gene Expression Omnibus (GEO) as GSE19754.

Principal component analysis (PCA) was used to survey gene variation across sample (time) and space, and sample variation across transcriptome space, separately.^21^ Because most of the time-points had replicate sample measurements, we computed the linear correlation between the unlogged replicate time profiles (A, B) for each probe set to assess the reproducibility of their time profile. We selected the probe set with the maximum replicate time profile correlation as the unique representative for genes with more than one probe set representative. The fold change of a probe set for days 10–14 vs. days 0–1 was computed as the average RMA signal of days 10–14 minus the average RMA signal of days 0–1. This fold change is in log base 2 scale, because the RMA signal is in log base 2 scale. Gene ontology (GO) enrichment analysis was performed using the Database for Annotation, Visualization, and Integrated Discovery (DAVID 6.7; http://david.abcc.ncifcrf.gov) on the mouse homologs of zebrafish genes, because the ontological characterization of genes is currently richer for the mouse than for the zebrafish.^22^ We used the mouse C2C12 myogenic differentiation microarray dataset (GEO, GSE19968) for comparative genomic analysis.^23^

### Quantitative Real-Time Polymerase Chain Reaction

Total RNA (1 μg) was extracted from the zebrafish muscle myogenic progenitor cells in culture at various time-points during differentiation and subjected to reverse transcriptase using the First Strand Synthesis Kit (Invitrogen). cDNA was then diluted in sterile water into tenfold serial dilutions, and real-time polymerase chain reaction (PCR) was performed (SYBR Green Master Mix; Applied Biosystems). Gene-specific primers that overlapped introns were used (refer to Supplementary Material, Table S5). All samples were amplified on a light cycler (Model 7900HT; ABI). Cycle time (CT) values were normalized to a zebrafish ef1α loading control. All significant values were determined using Student *t*-tests (two-tailed).

## RESULTS

### Isolation and Differentiation of Adult Zebrafish Myogenic Progenitor Cells

**FIGURE 1:**
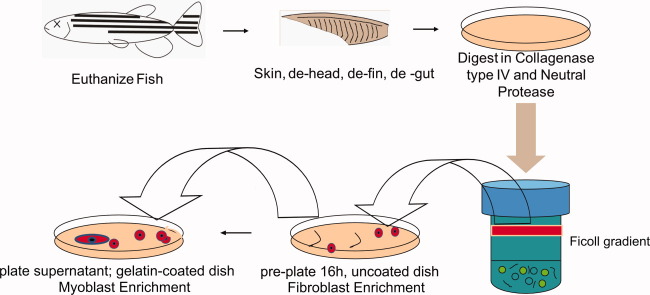
Basic protocol for the isolation of zebrafish skeletal muscle myogenic progenitor cells from whole dorsal myotome. Schematic showing the procedure for the isolation of skeletal myogenic progenitors from adult zebrafish dorsal muscle. Following euthanization of the zebrafish with tricaine, the fish are skinned, decapitated, de-finned, and de-gutted. A disassociation step in a mixture of collagenase IV and neutral protease breaks down cellular adhesion, whereas the use of a Ficoll gradient results in the isolation of a mononuclear cell layer. Pre-plating on uncoated plates was followed by an overnight (16-hour) transfer of the myoblast-enriched supernatant to gelatin-coated plates. [Color figure can be viewed in the online issue, which is available at wileyonlinelibrary.com.]

**FIGURE 2:**
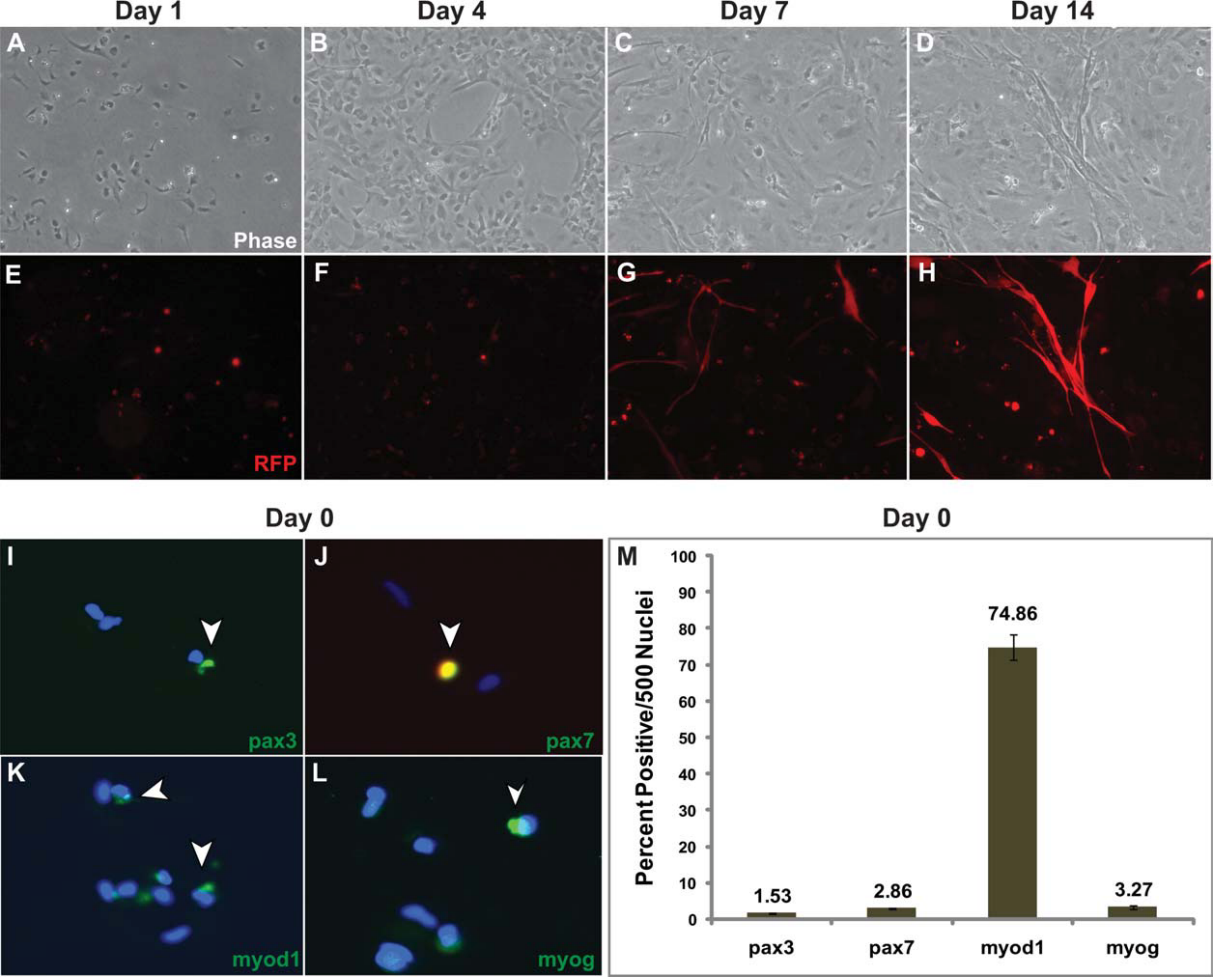
In vitro differentiation of primary myoblasts isolated from α-actin–RFP adult dorsal muscle. **(A–D)** Phase contrast of zebrafish myogenic progenitor cells differentiating from day 0 to day 14. **(E–H)** RFP expression of the α-actin promoter indicates myotube formation and myogenic differentiation. **(I–L)** Immunofluorescent staining of day 0 α-actin-–RFP myoblasts. Note that very few cells express high levels of the α-actin RFP transgene, as it undergoes higher levels of transcriptional expression during myogenic differentiation. Green fluorescent staining and open arrowheads demarcate myogenic markers (pax3, pax7, myod1, and myogenin). **(M)** Quantification of 500 DAPI-stained (blue) nuclei of the results from day 0 myoblast immunofluorescent staining in **(I)–(L)**. Immunostaining was performed in triplicate. [Color figure can be viewed in the online issue, which is available at wileyonlinelibrary.com.]

In mammals, it is possible to identify muscle progenitor cells by their potential to differentiate into multinucleate myotubes in culture. To access this capability in adult zebrafish, myogenic progenitor cells were prepared from α-actin–RFP transgenic zebrafish, as outlined in Figure 1 and detailed in the previous section. Following cellular expansion, after reaching 95%^+^ confluency (after being plated at 300,000 cells 24 hours earlier), the myogenic progenitor cells were exposed to differentiation medium. Over the course of 14 days, cultured zebrafish muscle cells began to fuse and elongate (Fig. 2). The use of the α-actin–RFP transgenic line allowed for the easy identification of mature myotubes in contrast to any few remaining fibroblasts due to the skeletal muscle-specific enhancer that drives expression of the RFP reporter, as characterized elsewhere.^17^

Initial plating of primary myoblasts from the α-actin–RFP fish resulted in very few RFP-positive cells either attached to the plates or free floating in the medium (Fig. 2A–H). At day 4, several clusters of RFP-positive cells emerged as the myoblasts began to undergo cellular fusion. The detection of RFP (α-actin) reporter was a strong indicator that the zebrafish myogenic progenitors had begun to activate transcripts essential for myoblast fusion and myotube structure, as the α-actin gene (promoter for RFP) expression is most robust in mature myofibers.^17^, ^24^ By day 7, long multinucleated RFP^+^ myotubes were identified that further expanded into twitching myotube clusters by day 14 (Fig. 2H).

To further characterize what stage of myogenesis these adult zebrafish myogenic progenitors resided in at the initial time of isolation (day 0), the cells were probed using immunofluoresence with the myogenic determination markers pax3 and pax7. Mammalian pax3 and pax7 function as determinants of the transition from embryonic myoblasts into muscle satellite cells, whereas, in zebrafish, these proteins function in the determination of fast muscle fibers used for swimming.^11^ Day 0 zebrafish myogenic progenitor cells had low levels of pax3 (1.53%) and pax7 (2.86%) protein expression, as quantified by immunofluorescence with monoclonal specific antibodies (Fig. 2I, J, and M). Conversely, these day 0 myogenic progenitors had significant levels of myod1 (74.86%), indicating that these cells were further committed than mammalian satellite cells to form myotubes (Fig. 2K and M). In addition, these cells had low expression of myogenin (3.27%) (Fig. 2L and M), a marker of myofiber determination. These experiments demonstrate that isolated myogenic progenitor cells can successfully fuse in cell culture as visualized by the α-actin–RFP fluorescent reporter, similar to the myoblast culture of larger fish species, such as the Atlantic salmon.^13^

### Transcriptome Profiles of Cell Fusion and Differentiation of Zebrafish Myogenic Progenitor Cells

To identify the myogenic transcriptome of zebrafish myogenic progenitor cells from cell proliferation through cell fusion and differentiation into mature myotubes, total mRNA was interrogated by microarray at different time-points (days 0, 1, 4, 7, 10, and 14) from zebrafish myogenic progenitor cells of the α-actin–RFP transgenic line as the cells underwent myogenic differentiation in culture.

**FIGURE 3:**
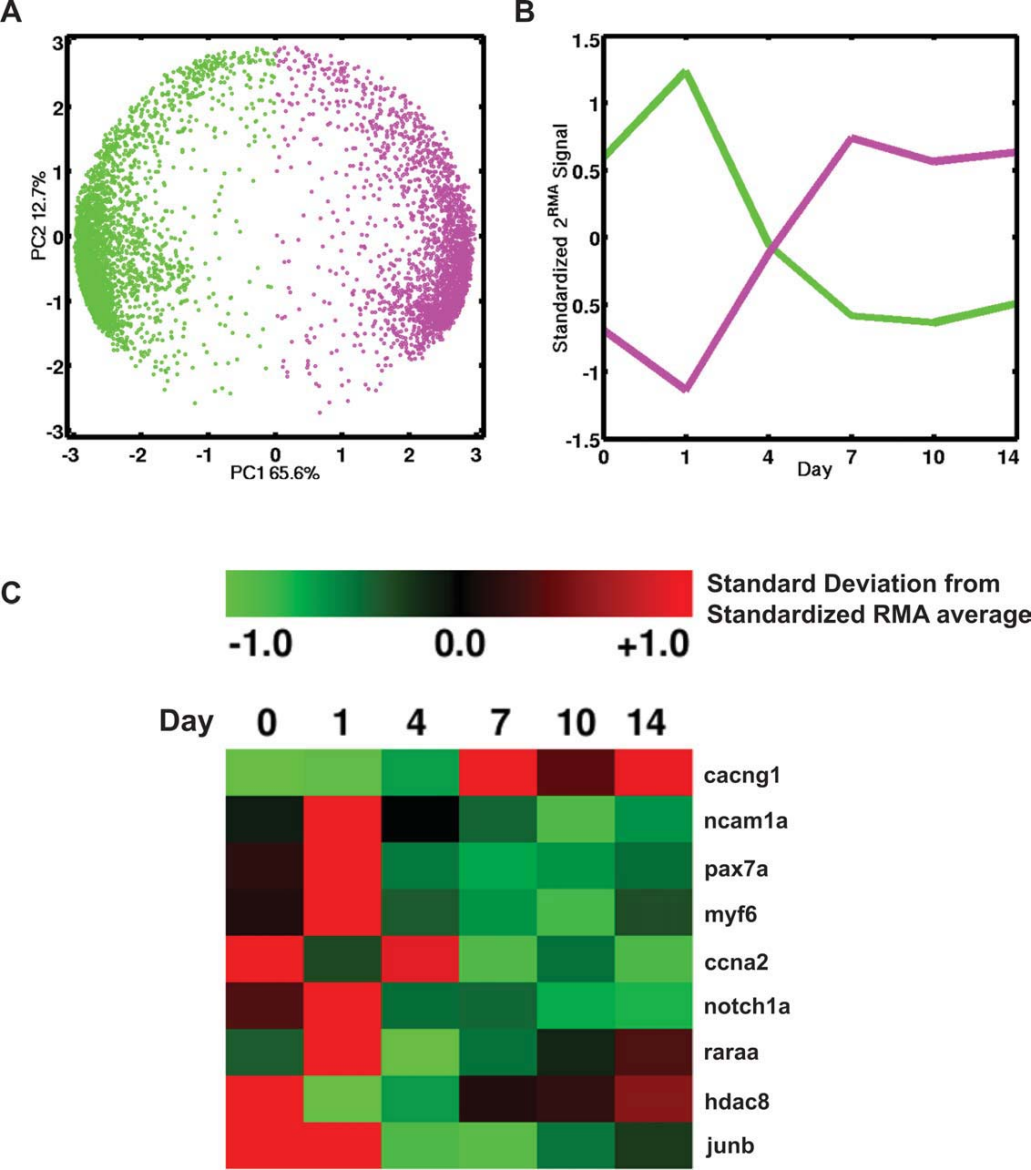
Microarray analysis of zebrafish myogenic progenitor cell differentiation transcriptome. **(A)** Principal components analysis (PCA) showing the principal components 1 vs. 2 plot of the zebrafish muscle cell differentiation microarray data of 5960 reproducible genes (shown as colored dots) in time and indicates two large-scale temporal patterns of expression. Genes on the left hemisphere (green) are highly expressed at days 0–1, and decrease over time. Genes on the right hemisphere (magenta) show low expression at days 0–1, and increase over time. The principal components axes are a linear combination of the time-points. **(B)** The average expression profile of the genes from the two large-scale temporal patterns of expression. **(C)** Standardized expression for upregulation (red) vs. downregulation (green) of nine differentially regulated myogenic genes. [Color figure can be viewed in the online issue, which is available at wileyonlinelibrary.com.]

Duplicate biological measurements (A, B) were made for most time-points. For each microarray gene probe set, we computed the correlation between duplicate profiles to assess the reproducibility of the myogenic developmental profile of the gene. There were 5960 microarray gene probe sets with a correlation >0.8 between duplicate profiles. Unless otherwise noted, this is the primary microarray gene set used in subsequent analyses. PCA of the standardized temporal expression profiles of these genes show them to have two large-scale temporal patterns (Fig. 3). Fifty-six percent (3340 genes, 2985 unique) have a profile that largely decreases with time (green dots, left hemisphere of PCA plot in Fig. 3A) and are enriched for development and cell signaling receptor ontologic terms (Supplemental Material, Table S1). Forty-four percent (2620 genes, 2414 unique) have a profile that is largely increasing with time (magenta dots, right hemisphere of PCA plot in Fig. 3A) and are enriched for oxidoreductive and metabolic enzyme ontologic terms (Supplementary Material, Table S2). The majority of genes change their expression level at day 4: high to low, and vice versa (Fig. 3B). Phenotypically, zebrafish muscle cells at day 4 of myogenic differentiation are in the initial stages of myotube fusion. To identify the active genes at day 4, we performed a differential analysis of day 4 vs. the other days (0, 1, 7, 10, and 14). Forty-seven unique genes were significantly upregulated at day 4 relative to the other days and were enriched for M-phase and mitosis ontologic terms (Supplementary Material, Table S3). Sixty unique genes were significantly downregulated at day 4 relative to the other days and were enriched for collagen and extracellular matrix ontological terms (Supplementary Material, Table S4). In addition, we examined the microarray expression profile of nine reproducible transcripts that have been reported previously to be differentially expressed during myogenesis.^25^

### Zebrafish Myogenic Progenitor Cells Express Myogenic Genes at Critical Time-Points during Differentiation

**FIGURE 4:**
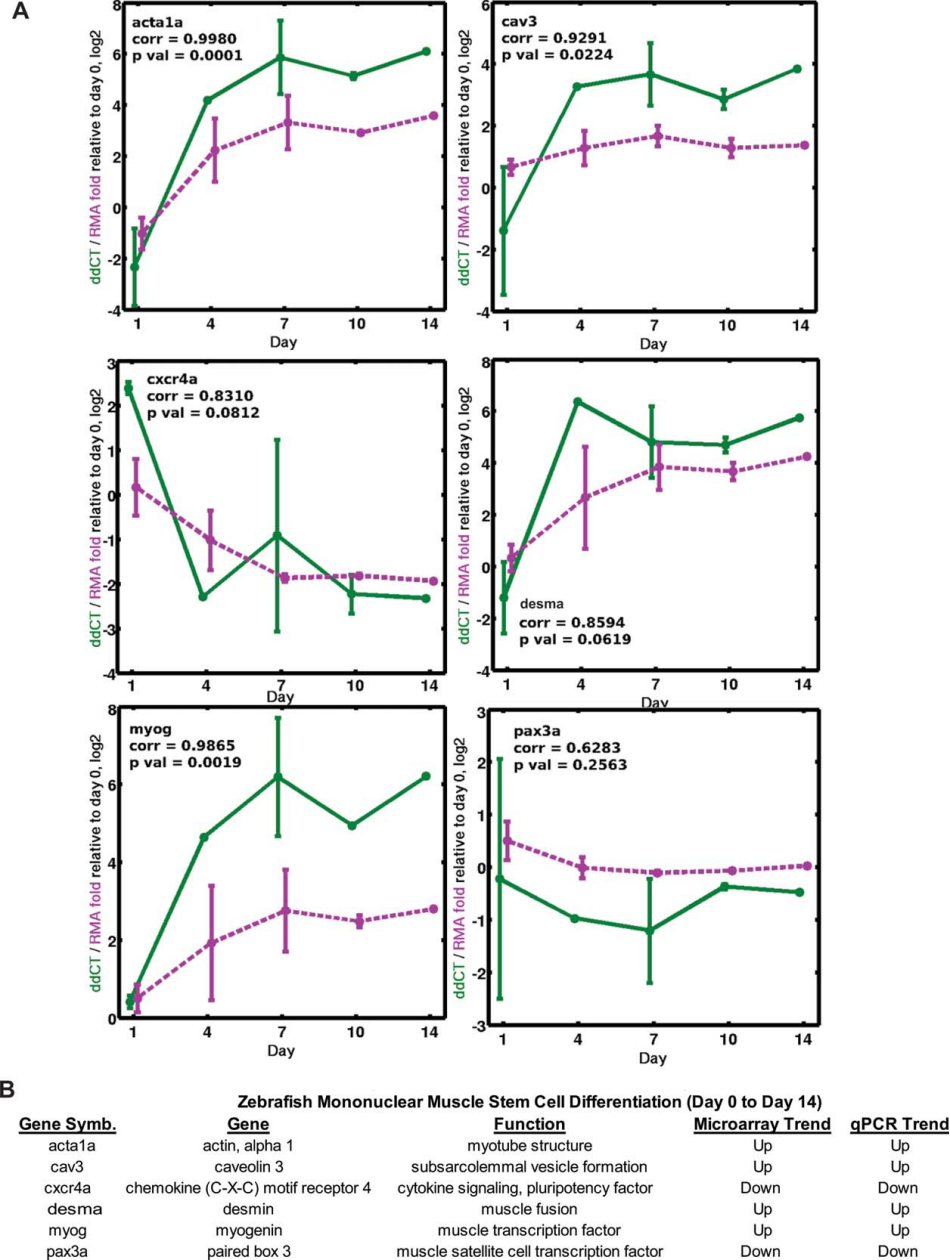
Validation of myogenic differentiation in the zebrafish myogenic progenitor cells by microarray and real-time PCR. **(A)** Real-time quantitative PCR expression (magenta dashed line) levels of six myogenic differentiation factors (*acta1a*, *cav3*, *cxcr4a*, *desma*, *myog*, and *pax3a*) across time (x-axis; days 0–14) as compared with microarray data (green solid line). The y-axis is logarithm base 2 scale fold change of each time-point relative to day 0, which is the average ΔCT (day 0) minus average ΔCT (day N) value for quantitative PCR data (ddCT), and average RMA signal (day N) minus average RMA signal (day 0) for the microarray data. The quantitative PCR CT values were normalized to the zebrafish housekeeping gene *ef1α* housekeeping per condition. Note that *acta1* and *cxcr4* primers were specific to both a and b isoforms present in the zebrafish genome. **(B)** The table compares the log2 expression fold change of days 0–1 vs. 10–14 of the six myogenic differentiation factors between quantitative PCR and microarray data. [Color figure can be viewed in the online issue, which is available at wileyonlinelibrary.com.]

After myogenic progenitor cell microarray analysis of the zebrafish, samples were validated by quantitative real-time PCR for several important myogenic genes using exon-overlapping primers. Several myogenic structural (*acta1a*, *desma*), cell-signaling (*cav3*, *cxcr4a*), and transcription (*myog*, *pax3a*) factors were chosen for validation. In each case, each gene followed the expected microarray trend across myogenic differentiation (Fig. 4). The myogenic structural genes (*acta1a* and *desma*) were all upregulated as the zebrafish myogenic progenitor cells underwent myogenic fusion and myotube formation. As expected, the myogenic stem cell marker (*cxcr4a*) mRNA was downregulated as the zebrafish muscle cells underwent fusion, whereas, conversely, the myogenic transcription factor myogenin (*myog*) was upregulated. In addition, another marker of early myoblasts, *pax3a*, had significantly reduced expression as the cells underwent myogenic differentiation.

### Comparison of Zebrafish Myogenic Progenitor Cell Transcriptome with other Mammalian Myogenic Transcriptomes: Strengths and Limitations

**FIGURE 5:**
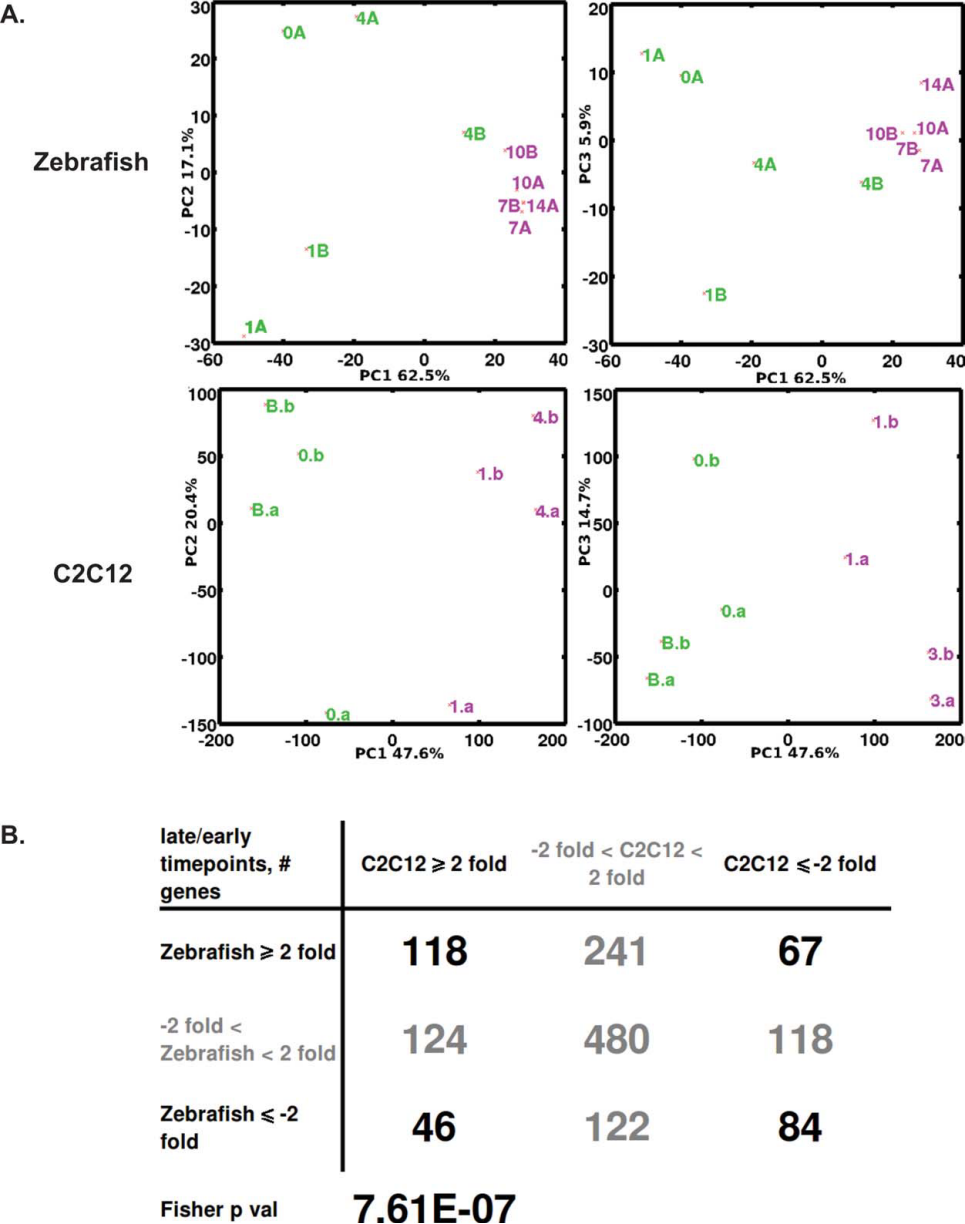
Comparison of zebrafish and mouse C2C12 myogenic development. **(A)** Principal components analysis of samples in transcriptome space showing principal components 1 vs. 2, and 1 vs. 3 plots for the zebrafish and C2C12 (from Gene Expression Omnibus, GSE19968) data show transcriptome scale distinctions between earlier vs. later time-points of muscle development: days 0–1 vs. days 7–10 in zebrafish, and myoblasts vs. differentiated myotubes at day 4 in C2C12. Zebrafish samples are labeled by the time-point following myogenic differentiation (days 0–14). C2C12 samples are labeled as myoblasts **(B)**, and time-points following myogenic differentiation (days 0, 1, and 4). **(B)** Contingency table of genes ≥2-fold magnitude changed in earlier vs. later time-points of 1400 reproducible genes common to both datasets: fold change of days 10–14 relative to days 0–1 in zebrafish, and fold change of myotubes at day 4 relative to myoblasts in C2C12. [Color figure can be viewed in the online issue, which is available at wileyonlinelibrary.com.]

To gain insights into similarities between zebrafish and mammalian myogenic cells with respect to changes in gene expression during in vitro differentiation, we compared the zebrafish myogenic differentiation transcriptome data to a recent mouse C2C12 myogenic differentiation microarray dataset from the GEO, GSE19968.^23^ PCA of samples in transcriptome space of both datasets, done separately, showed a distinct dichotomy between the earlier vs. later time-points of myogenic differentiation along the first principal component (PC1), the direction of maximum sample variation (Fig. 5A). There is a clear transcriptome scale distinction when comparing days 0–1 vs. days 7–10 in the zebrafish, and between myoblasts and differentiated myotubes at day 4 in C2C12. There are 3784 homologous genes in common between the datasets, and 1400 have a correlation of >0.8 between replicate time profiles in both datasets, respectively. Of these 1400 reproducible genes, we investigated the concordance of differential expression of earlier vs. later time-points during myogenic differentiation. We computed the fold change of days 10–14 relative to days 0–1 in the zebrafish, and of myotubes at day 4 relative to myoblasts in C2C12. There was significant concordance among genes that were twofold magnitude changed at earlier vs. later time-points in both datasets: Fisher exact test *P*-value <7.0 × 10^−7^ (Fig. 5B).

## DISCUSSION

Gene expression profiles of early differentiating zebrafish myogenic progenitor cells show expression profiles similar to those expected for mammalian muscle, namely that the expression of many sarcomeric proteins is strongly upregulated with differentiation. In comparison with microarray data from mouse C2C12 myoblast differentiation,^25^ many of the same myogenic differentiation factors, such as Pax3, Myf5, and MyoD1, decrease in transcript. Although Pax3 is a determinant of embryonic mouse myoblasts, recent studies involving the use of Pax3–green fluorescent protein (GFP) knock-in mice have revealed that a very small population of Pax3-positive myogenic progenitors does persist in adult muscle and are capable of restoring skeletal muscle after injury.^26^ In zebrafish dorsal muscle, a *pax3*- and *pax7*-positive myogenic progenitor population is essential for the expansion of fast- and slow-twitch myofibers through an upstream regulation of *myf5* and *myod1*.^27^ It is likely that a similar population of *pax3*- and/or *pax7*-positive myogenic progenitors exists in adult zebrafish skeletal muscle, and will contribute to myofiber formation following injury. In addition, the presence and subsequent downregulation of a *cxcr4a* (a homolog of mammalian Cxcr4) cell population during myogenic differentiation is consistent with its role as a myogenic progenitor marker that can be used in myoblast transplantation.^28^ A transparent zebrafish strain that completely lacks pigmentation, the *casper* line, allows for the transplantation and long-term monitoring of fluorescently labeled cell populations into adult fish. One can envision that, after the isolation of adult zebrafish myogenic progenitor cells from skeletal muscle transgenic fish lines, engraftment of different populations could be observed in vivo, allowing for the capture in real time of the behavior of transplanted cells. This information is essential for the optimization of cell transplantation approaches (now available with the development of this zebrafish myogenic progenitor isolation protocol) which cannot be visualized in mice at the level of resolution that can be achieved in zebrafish.

In mice, many procedures have been used to purify muscle progenitor cells, although, in all cases, the purified population is still heterogeneous, requiring additional pre-plating purification to enrich for cells with myogenic potential.^16^ We have modified the mammalian pre-plating technique, added a Ficoll-gradient procedure to decrease bacterial contamination, and demonstrated that myogenic cells can be isolated and differentiated in cell culture. These results show that zebrafish have adult muscle progenitor cells that can be isolated and differentiated in cell culture.

In conclusion, we have isolated a myogenic progenitor cell in zebrafish dorsal muscle. We have shown that gene expression profiles in these zebrafish myogenic progenitor cells are similar to those of mammals. This successful culture and differentiation of a myogenic progenitor population expands the utility of the zebrafish in the study of adult skeletal muscle mutants. High-throughput screening of chemical libraries has allowed researchers to correct mutations in zebrafish mutants and holds promise for the treatment of muscular dystrophy and myopathies.^29^ Given the gaps in the zebrafish genome annotation that have frustrated researchers,^30^ it is likely that the release of a well-annotated copy of the zebrafish genome will lead to improved microarray platforms and increased use of the zebrafish in large-scale transcriptome studies. Until then, rigorous validation of zebrafish transcriptome data by quantitative reverse transcription PCR is essential for drawing valid conclusions from zebrafish microarray transcriptome experiments. Further studies using zebrafish myogenic progenitor cells to identify novel drug compounds will show them to be an attractive, cost-effective alternative to large-scale mammalian studies.

## Abbreviations

CT: cycle time
DAPI: 4′,6-diamidino-2-phenylindole
DAVID: Database for Annotation, Visualization, and Integrated Discovery
DMEM: Dulbecco's modified Eagle medium
GEO: Gene Expression Omnibus
GFP: green fluorescent protein
PBS: phosphate-buffered saline
PCA: principal components analysis
PCR: polymerase chain reaction
RFP: red fluorescent protein
rhFGF: recombinant human fibroblast-like growth factor
RMA: robust multiarray averaging
